# Twitter-Based Analysis of the Dynamics of Collective Attention to Political Parties

**DOI:** 10.1371/journal.pone.0131184

**Published:** 2015-07-10

**Authors:** Young-Ho Eom, Michelangelo Puliga, Jasmina Smailović, Igor Mozetič, Guido Caldarelli

**Affiliations:** 1 IMT Institute for Advanced Studies, Piazza San Francesco 19, 55100 Lucca, Italy; 2 Jožef Stefan Institute, Jamova 39, 1000 Ljubljana, Slovenia; 3 Istituto dei Sistemi Complessi (ISC), via dei Taurini 19, 00185 Roma, Italy; 4 London Institute for Mathematical Sciences, 35a South Street Mayfair, London, W1K 2XF, UK; 5 Linkalab, Complex Systems Computational Laboratory, Cagliari, Italy; University of Maribor, SLOVENIA

## Abstract

Large-scale data from social media have a significant potential to describe complex phenomena in the real world and to anticipate collective behaviors such as information spreading and social trends. One specific case of study is represented by the collective attention to the action of political parties. Not surprisingly, researchers and stakeholders tried to correlate parties' presence on social media with their performances in elections. Despite the many efforts, results are still inconclusive since this kind of data is often very noisy and significant signals could be covered by (largely unknown) statistical fluctuations. In this paper we consider the number of tweets (tweet volume) of a party as a proxy of collective attention to the party, identify the dynamics of the volume, and show that this quantity has some information on the election outcome. We find that the distribution of the tweet volume for each party follows a log-normal distribution with a positive autocorrelation of the volume over short terms, which indicates the volume has large fluctuations of the log-normal distribution yet with a short-term tendency. Furthermore, by measuring the ratio of two consecutive daily tweet volumes, we find that the evolution of the daily volume of a party can be described by means of a geometric Brownian motion (i.e., the logarithm of the volume moves randomly with a trend). Finally, we determine the optimal period of averaging tweet volume for reducing fluctuations and extracting short-term tendencies. We conclude that the tweet volume is a good indicator of parties' success in the elections when considered over an optimal time window. Our study identifies the statistical nature of collective attention to political issues and sheds light on how to model the dynamics of collective attention in social media.

## Introduction

As social animals, since a long time ago, humans have communicated, exchanged opinions, and tried to reconcile their conflicts by means of social instruments. Despite their recent introduction, social media and web-based services such as Google, Twitter, Facebook, and Wikipedia have already dramatically changed the way in which people make relationships, interact with others, and acquire information. Differently from the past, such activities help people to overcome the physical and geographical limitations of human interactions.

When people use social media and web services, a huge amount of digital “footprints” (i.e., data) are created and simultaneously recorded. These “footprints” can provide us novel opportunities to observe collective behaviors at unprecedented scales. For this reason, the data are generally regarded as crucial instruments in order to understand the complex and collective behaviors in our social and technological systems [1–5]. Despite the recent appearance of these computer-based social media, there is already a large number of studies describing and forecasting collective behaviors emerging from them. For example, large scale network analysis based on Twitter and Facebook data have revealed the structure of social networks of tens of millions of people [6, 7]. Twitter data have been used to identify spreading patterns of popular information [8, 9], classes of dynamical collective attention [10], linguistic usage patterns on worldwide scale [11], and political activity [12–14]. From Facebook data it has been possible to distinguish difference in consumption patterns between science and conspiracy information [15]. Further cross-cultural differences in evaluation of historical figures were identified based on multilingual Wikipedia data [16, 17], and social media usage patterns are used to find out unemployment in local regions [18]. Finally, users’ query logs on search engines help to anticipate the spreading of flu [19] or dynamics of stock market [20, 21], and Wikipedia activity data was used to predict movies’ box office [22].

Predictions of elections based on social media data have various advantages with respect to other methods (such as traditional opinion polls). Firstly, we deal with large scale samples, secondly, the flow of data is such that we can get real time responses, and finally, we have low costs of data collection. For these reasons, social media data received (and probably will receive even more in the future) a great attention by practitioners and scientists. The key question will be whether relevant information on elections can be extracted from social media data or not. It is now known that in certain cases we can have indications on elections results, but the degree of reliability of this method has to be improved [23]. For example, both positive [24–27] and null relations [28, 29] between social media activity and election outcomes have been observed so far. In order to improve this method of forecast, some scientists suggest to complement tweet volume analysis with sentiment analysis of tweets, i.e., identification of positive or negative sentiment [27, 30]. Nevertheless, reliable methods of sentiment analysis for political tweets are still lacking [31]. Intuitively, mentions of political parties or politicians in social media can be considered as expressions of people’s attention to them. However, there is no guarantee that all of the mentions in social media correspond to the supports for the parties in elections. People post tweets on political parties and politicians for various reasons, such as expressions of support, disappointment, or sarcasm. In other words, dynamics of tweet activity can be driven not only by popularity of parties or politicians but also by other reasons. Therefore it is necessary to understand dynamics of collective attention to political parties or politicians in social media, since such understanding will be a cornerstone to separate the “signal” from the “noise” in the dynamics of collective attention in social media.

In this paper we consider tweet volumes about political parties as proxies of collective attention to the parties and by investigating the dynamics of tweet volumes we try to assess their relation (and forecasting power) with the final results of elections. For such purposes, we identify dynamical and statistical characteristics of daily tweet volumes of political parties during election periods. We find that the distributions of daily tweet volume of each political party is in good agreement with log-normal distribution [32]. This observation indicates that the average behavior of daily tweet volume may have some information, yet large fluctuations can be behind the average. Thus the prediction based on too short-term Twitter data may not be consistent. On the other hand, we observed positive autocorrelation of daily tweet volume of each party in short term. This means the time series of daily tweet volume largely depends on the previous activity (i.e., the existence of short-term tendency). Thus, averaging over too long-term periods can destroy the signal. We also measure that the distribution of the logarithmic ratio of two consecutive daily tweet volumes for each party follows a normal distribution and the ratio is independent of time. These two observations allow us to describe properly the dynamics of daily tweet volume as a geometric Brownian motion [33]. In the end, we checked whether there is an optimal period of averaging tweet volumes which not only reduce the fluctuation but also keep the short term tendency of tweet volumes. Our analysis suggests what really tweet volume of each political party means in a quantitative way and sheds light on how we can separate the noise and the signal for better prediction using social media data.

## Materials and Methods

### Data description

In this paper, we consider data collected on Twitter (twitter.com), a microblogging platform used by millions of bloggers. In Twitter, each user can freely post short messages (up to 140 characters) called “tweets” to its followers. Twitter provides application programming interfaces (APIs) to access tweets and information about tweets and users. The potential bias of Twitter APIs was discussed by a recent research [34]. We mainly consider daily tweet volume *V*_*p*_(*t*) of a given political party *p* at day *t*. To identify dynamics of daily tweet volume of political parties in Twitter, we consider three elections in two European countries: *European Parliament election of 2014 in Italy (Euro14)*, *Italian general election of 2013 (Italy13)*, and *Bulgarian general election of 2013 (Bulgaria13)*. By using Twitter API, we collected general tweets around election days and then considered only tweets posted in local languages (i.e., Italian or Bulgarian) from the starting day of data collection to the day before the election day. We used the implemented automatic language detection system of Twitter to identify the language of tweets. For the Bulgarian case, the Twitter language detection mechanism often did not distinguish between Bulgarian and Macedonian, which are very similar. We therefore implemented our own language detection, based on a Bayesian classifier, trained on a large corpus of over five million words for each language. Here one day is defined as a time window from 00:00:00 to 23:59:59 of the day in local time for the Italian cases and Greenwich Mean Time for the Bulgarian case. For the cases of election in Italy (i.e., *Euro14* and *Italy13*), we define the number of tweets *V*_*p*_(*t*) for a given political party *p* as the number of tweets mentioning the leaders’ names (only family names) of political parties *p* or the leaders’ twitter accounts at the day *t*. This is because, in Italian cases, the names of leaders are widely used to represent the political parties [26]. The overview summary of three data sets are represented in Table 1.

**Table 1:** Description of Twitter data set. Time stamps in *Euro14* and *Italy* are in local time while time stamps in *Bulgaria13* are in Greenwich Mean Time (GMT). There is a three-hours difference between GMT and Bulgarian time. *T*_*i*_ represents the initial day of considered data. *T*_*e*_ is the election day. *T*_*f*_ represents the final day of considered data. One-day is defined a time interval from 00:00:00 to 23:59:59 in considered time. *N*_*T*_ represents the total number of considered tweets for given time interval from *T*_*i*_ to *T*_*e*_-1 posted in local language. *N*_*P*_ represents the number of considered political parties.

**Data set**	*T*_*i*_	*T*_*e*_	*T*_*f*_	*N*_*T*_	**Language**	*N*_*P*_	**Held in**
*Euro*14	22 Apr. 2014	25 May 2014	12 Jun. 2014	3,413,214	Italian	7	Italy
*Italy*13	1 Jan. 2013	23 Feb. 2013	3 Mar. 2014	3,796,754	Italian	6	Italy
*Bulgaria*13	29 Apr. 2013	12 May 2013	27 May 2013	5,817	Bulgarian	4	Bulgaria

- *European Parliament election of 2014 in Italy (Euro14)*: We collected 12,535,469 tweets posted between 21 April 2014 and 12 June 2014 in total. Of this sample, we extracted 3,413,214 Italian tweets between 22 April 2014 and 23 May 2014. The election day was 24 May 2014 [35].
- *Italian general election of 2013 (Italy13)*: We collected 7,755,063 tweets posted between 11 November 2012 to 3 March 2013 in total. Of this sample, we extracted 3,796,754 Italian tweets from 1 January 2013 to 22 February 2013. The election days were 23 and 24 February 2013 [36].
- *Bulgarian general election of 2013 (Bulgaria13)*: The raw tweet data is based on collected 16,077 tweets posted between 29 April 2013 to 27 May 2013 in total [27]. Out of this sample, we extracted 5,817 tweets from 29 April to 11 May 2013. The election day was 12 May 2013 [37]. In this case we consider both, the names of political parties and the names of their leaders. The retrieval of the Bulgarian tweets was performed by the Gama System company (http://www.gama-system.si/en/) and their Gama System® PerceptionAnalytics platform (http://demo.perceptionanalytics.net)

Detailed information on each party in each election is given in Table 2.

**Table 2:** Description of considered political parties for each election. The official sources of election results are provided on [35](*Euro14*), [36](*Italy13*), and [37] (*Bulgaria13*) respectively.

**Euro14: European Parliament election 2014, Italy**			
**Rank**	**Party**	**Actual votes**	**Leaders**
1	Partito Democratico (PD)	11,203,231	Matteo Renzi
2	MoVimento Cinque Stelle (M5S)	5,807,362	Beppe Grillo
3	Forza Italia (FI)	4,614,364	Silvio Berlusconi
4	Lega Nord (LN)	1,688,197	Matteo Salvini
5	Nuovo Centrodestra—Unione di Centro (NCD-UdC)	1,202,350	Angelino Alfano, Pier Ferdinando Casini
6	L’Altra Europa con Tsipras (AET)	1,108,457	Alexis Tsipras, Nichi Vendola, Paolo Ferrero
7	Fratelli d’Italia—Alleanza Nazionale (FdI-AN)	1,006,513	Giorgia Meloni
**Italy13: Italian general election 2013**			
**Rank**	**Party**	**Actual votes**	**Leaders**
1	MoVimento Cinque Stelle (M5S)	8,691,406	Beppe Grillo
2	Partito Democratico (PD)	8,646,034	Pier Luigi Bersani, Matteo Renzi
3	Il Popolo della Libertà (PdL)	7,332,134	Silvio Berlusconi
4	Scelta Civica (SC)	2,823,842	Mario Monti
5	Lega Nord (LN)	1,390,534	Roberto Maroni
6	Sinistra Ecologia Libertà (SEL)	1,089,231	Nichi Vendola
**Bulgaria13: Bulgarian general election 2013**			
**Rank**	**Party**	**Actual votes**	**Leaders**
1	GERB	1,081,605	Boyko Borisov
2	BSP	942,541	Sergei Stanishev
3	DPS	400,446	Lyutvi Mestan
4	ATAKA	258,481	Volen Siderov

### Geometric Brownian motion

Defining a geometric Brownian motion for the daily tweet volume *V*_*p*_(*t*) (for a party *p*) means that *V*_*p*_(*t*) satisfies the following stochastic differential equations [33, 38]:

$$dV_{p}\left(t\right)=\mu V_{p}\left(t\right)dt+\sigma V_{p}\left(t\right)dW_{t}$$ (1)

where *W*_*t*_ is Wiener process or Brownian motion, and *μ* and *σ* are constants. In particular, *μ* represents the “drift” (i.e., trend) and *σ* represents the “volatility” (i.e., random noise) of *V*_*p*_(*t*). Eq 1 has an analytic solution under Ito’s interpretation [39] as following:

$$V_{p}\left(t\right)=V_{p}\left(0\right)exp\left(\left(\mu -\frac{\sigma^{2}}{2}\right)t+\sigma W_{t}\right)$$ (2)

where *V*_*p*_(0) is the initial value.

Taking logarithm of both sides of Eq 2, we get:

$$log\left(V_{p}\left(t\right)\right)=log\left(V_{p}\left(0\right)\right)+\left(\mu -\frac{\sigma^{2}}{2}\right)t+\sigma W_{t}$$ (3)

Since ⟨*W*(*t*)⟩ = 0, the expectation value of *log*(*V*_*p*_(*t*)) is given in the following equation:

$$\left\langle log\left(V_{p}\left(t\right)\right)\right\rangle =log\left(V_{p}\left(0\right)\right)+\left(\mu -\frac{\sigma^{2}}{2}\right)t$$ (4)

## Results

The main results of this paper are summarized as follows. (i) We find that the daily tweet volumes of political parties before elections follow log-normal distributions and have positive autocorrelations over short terms. (ii) The daily volume evolution can be described by means of geometric Brownian motion. (iii) If we want to consider the average behavior of daily tweet volume, it is necessary to consider long enough period for reducing statistical fluctuations, but not too long, to not destroy short-term memories with relevant information.

### Indication from tweet volumes

**Fig 1:**
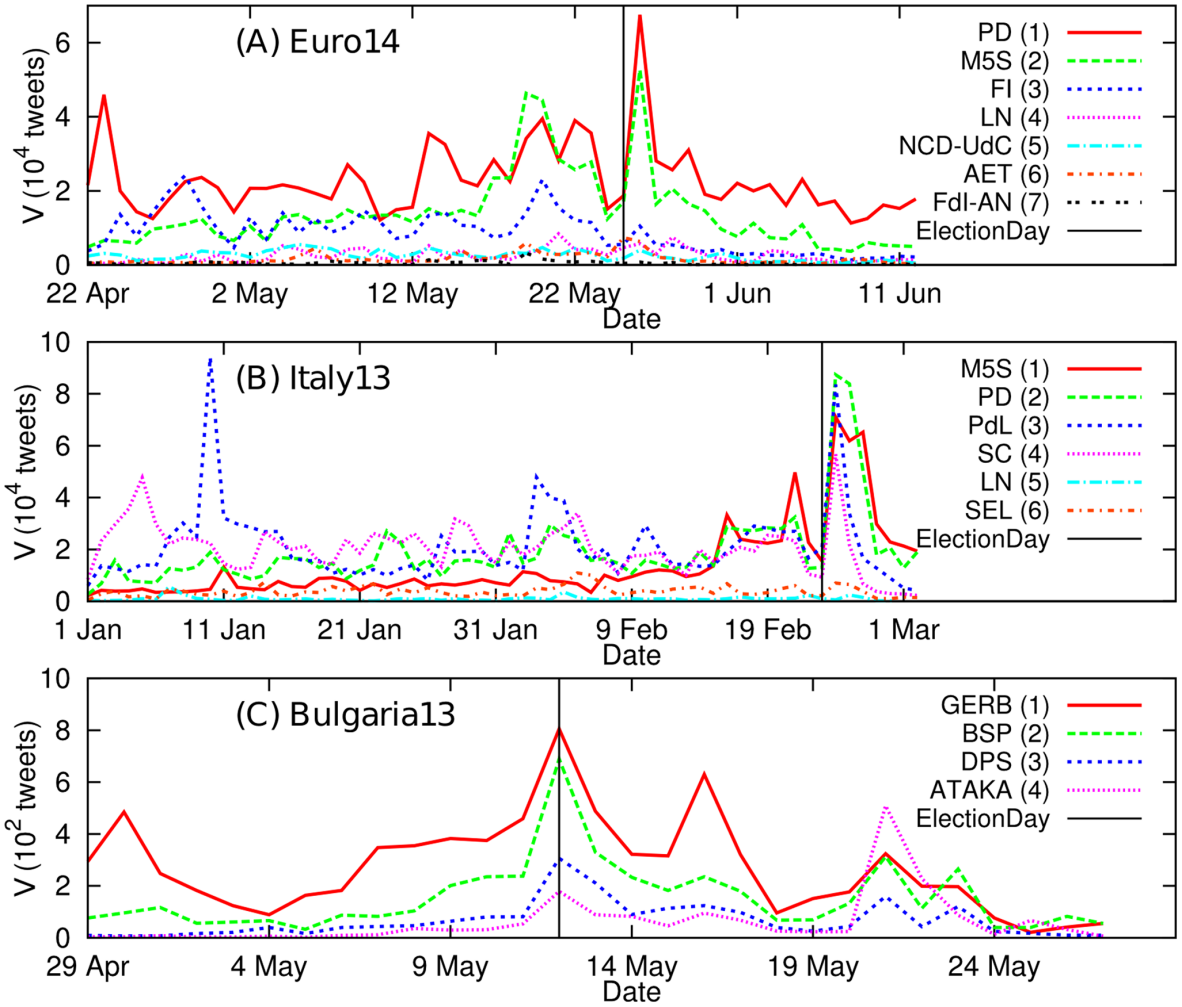
Daily tweet volume for each party around elections. The ordering of parties (i.e., the numbers in parentheses) is based on actual ranking in the election. (A) *Euro14*. 1st: PD. 2nd: M5S. 3rd: FI. 4th: LN. 5th: NCD-UdC. 6th: AET. 7th: FdI-AN. (B) *Italy13*. 1st: M5S. 2nd: PD. 3rd: PdL. 4th: SC. 5th: LN. 6th: SEL. (C) *Bulgaria13*. 1st: GERB. 2nd: BSP. 3rd: DPS. 4th: ATAKA.

We consider dynamics of daily tweet volumes of political parties in three elections (*Euro14*, *Italy13*, and *Bulgaria13*) based on the Twitter data collected as described in the Method section. The time series of daily tweet volume *V*_*p*_(*t*) of a political party *p*, before and after each election day, are represented in Fig 1. Sharp peaks of daily tweet volumes of parties on the election days and on the day after election days suggest the daily tweet volumes reflect the attentions of the public to the elections. On the other hand, other notable peaks are also observed much earlier than the election days, which indicate the daily tweet volumes may be activated by other reasons than election issues, such as scandals of politicians, their appearances in the press or mass media, or other political activities [40].

For these three election cases, we want to check if we can get an indication on the election outcomes simply considering daily tweet volume of parties or its simple functions as reported in some studies [24–26]. As shown in Fig 1, the daily tweet volume for each party shows different prediction power for election outcomes depending on elections. The ordering of parties in Fig 1 is determined by actual rankings based on number of votes in the elections (See Table 2). In the case of *Bulgaria13* (Fig 1(C)), during the whole observation period, rankings by the daily tweet volumes are the same as the actual election outcome. In the case of *Euro14* (Fig 1(A)), for most of observation days, daily tweet volume predicted well the election outcome. In the *Italy13* case (Fig 1(B)), the prediction is less effective than the other two cases especially in early days. In the *Italy13* case, the rankings predicted by analysis change frequently with the day, therefore making the forecast not very reliable. However, we cannot conclude that this is a failure of the method, since it could actually reflect the real dynamics of voters’ opinions. Indeed, according to the opinion polls in Italy [41], M5S had low support from the public in the early period of the campaign. Also it is notable that the *Italy13* case is a typical ‘too close to call’ case (See Table 2 for the actual number of votes) to evaluate the prediction power.

### Description of fluctuations in tweet volumes

The observed fluctuations in daily tweet volumes can distort not only prediction of parties’ rankings in elections but also the prediction on parties actual votes in the elections. While it seems possible to forecast rankings in some elections there is still some work to be done to anticipate the number of actual votes. Indeed, depending on the observation period, the prediction of the number of votes varied because strong fluctuations exist in daily tweets volumes for each party. Similar behaviors were also observed previously [24, 26].

**Fig 2:**
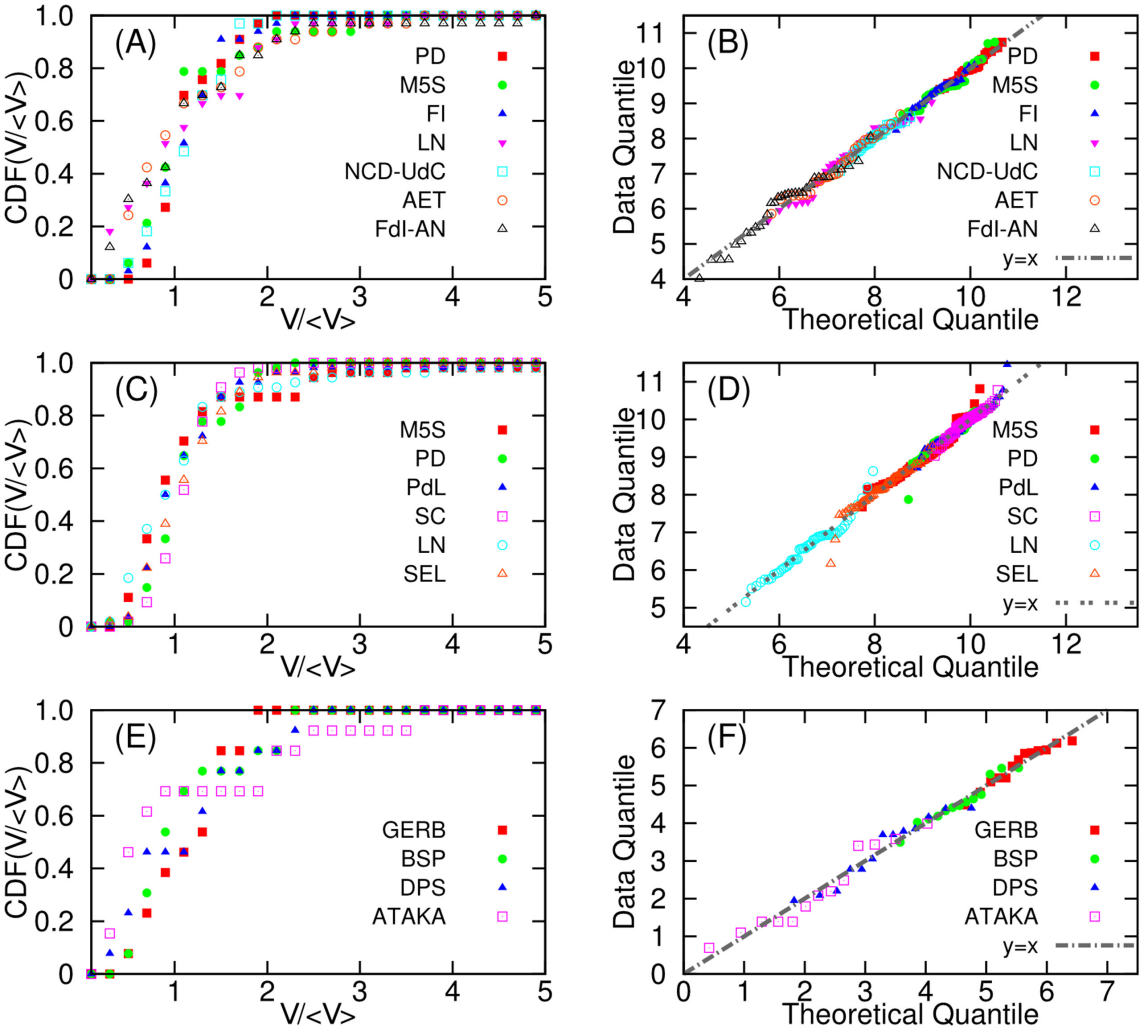
Cumulative distribution functions (CDF) of daily tweet volumes (A, C, E) and Q-Q plots of logarithms of daily tweet volumes for each political party (B, D, F). Each volume in CDF is normalized by the average ⟨*V*⟩. (A) CDF of daily tweet volume of *Euro14*. (B) Q-Q plot of *Euro14*. (C) CDF of daily tweet volume of *Italy13*. (D) Q-Q plot of *Italy13*. (E) CDF of daily tweet volume in *Bulgaria13*. (f) Q-Q plot in *Bulgaria13*. Note that Q-Q plot is for logarithm of daily tweet volume. Theoretical quantile in the Q-Q plot is based on normal distribution. Thus if the points in the Q-Q plot lie on *y* = *x* line, the daily tweet volume follows a log-normal distribution since the logarithm of the volume follow a normal distribution.

If the daily tweet volumes of parties show strong fluctuations, it is necessary at least to describe the statistical patterns of the evolution of this quantity. To this aim, we consider distributions of daily tweet volume *V*_*p*_ for the given time interval from the initial day of data collection to the day before the elections. From visual inspection, this quantity seem to follow a fat-tailed like “log-normal” distributions (Fig 2(A), 2(C), and 2(E)). Due to the small number of data samples, we represented the cumulative distribution functions. To determine whether the daily tweet volumes follows or not log-normal, we consider Q-Q plot (quantile-quantile plot) [38] of logarithm of *V*_*p*_ as shown in Fig 2(B), 2(D), and 2(F).

Note that if the points in the Q-Q plot are close to *y* = *x* line, the data is more likely to follow the theoretical distribution (i.e., normal distribution in this case). As shown in Fig 2(B), 2(D), and 2(F), in most of the cases we can conclude that the daily tweet volumes follow log-normal distributions since logarithms of the volumes follow normal distributions as shown in the Q-Q plots. Such fat-tailed shape means that even if the daily tweet volume may provide relevant information on the dynamics of collective attention to political issues, this information can be largely hidden by statistical fluctuations. Thus, in spite of some prediction power, it is not easy to predict the election outcome very accurately beyond the rankings due to the fluctuations.

We then checked whether the dynamics of the daily tweet volumes *V*_*p*_ can be described by a constant volume with fluctuations, or if there exist higher orders in the dynamics. First, in order to check if the daily tweet volumes can be described as a constant volume term with a noise volume term, we consider autocorrelation *R*_*p*_ of the daily tweet volume *V*_*p*_(*t*) for each party *p*. If we can consider *V*_*p*_(*t*) = *V*_0_ + *E*_*t*_, where *V*_0_ is a constant and *E*_*t*_ is an error term, *V*_*p*_(*t*) will move around *V*_0_ as a random signal without any short or long term tendency. In this case, autocorrelation of *V*_*p*_ will be zero. The autocorrelation measures how similar is the original time series of a variable to the lagged time series of the variable. We can measure autocorrelation *R*_*p*_(*τ*) of daily tweet volume for a party *p* with a lagged time *τ* by the Pearson’s coefficient between original tweet volume from day *t* = 0 to *t* = *t*_*e*_−1−*τ* and the same tweet volume from day *t* = *τ* to *t* = *t*_*e*_−1 for a given party *p* and *τ*:

$$R_{p}\left(\tau \right)=\frac{1}{t_{e}-\tau }\sum_{t=0}^{t_{e}-1-\tau }\frac{\left(V_{p}\left(t\right)-\left\langle V\right\rangle \right)\left(V_{p}\left(t+\tau \right)-\left\langle V^{\prime }\right\rangle \right)}{\sigma_{p}\sigma_{p}^{\prime }}$$ (5)

**Fig 3:**
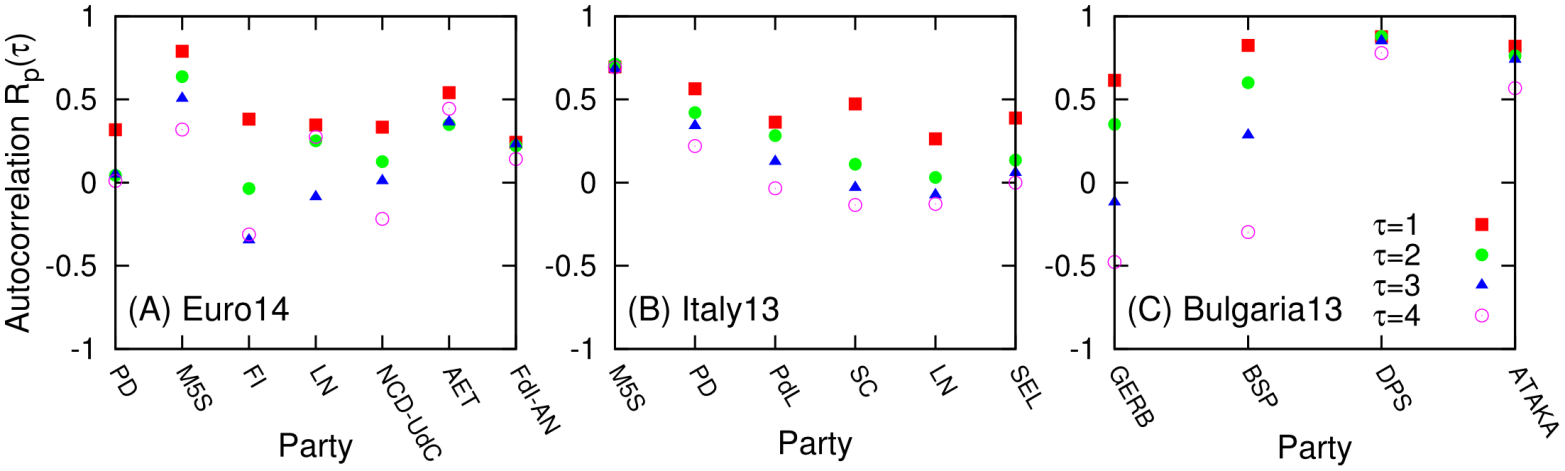
Autocorrelation of daily tweet volume for each political party. Autocorrelation coefficient *R*_*p*_(*τ*) is given by $R_{p}(\tau )=\frac{1}{t_{e}-\tau }\sum_{t=0}^{t_{e}-1-\tau }\frac{\left(V_{p}(t)-\langle V\rangle )(V_{p}(t+\tau )-\langle V^{\prime }\rangle \right)}{\sigma_{p}\sigma_{p}^{\prime }}$. Here ⟨*V*⟩ (⟨*V*′⟩) is the average daily tweet volume for party *p* from day *t* = 0 (*t* = *τ*) to day *t* = *t*_*e*_−1−*τ* (*t* = *t*_*e*_−*τ*), *σ*_*p*_ ($\sigma_{p}^{\prime }$) is the standard deviation, and *t*_*e*_ is the election day. (A)*Euro14*. (B) *Italy13*. (C) *Bulgaria13*.

Here, 〈*V*〉 (〈*V*′〉) is the average daily tweet volume for party *p* from day *t* = 0 (*t* = *τ*) to day *t* = *t*_*e*_−1−*τ* (*t* = *t*_*e*_−1), *σ*_*p*_ ($\sigma_{p}^{\prime }$) is the standard deviation, and *t*_*e*_ is the election day. Thus *R*_*p*_(*τ*) quantifies the correlation between original time series of daily tweet volume *V*_*p*_(*t*) with *τ* day-lagged time series *V*_*p*_(*t* + *τ*) of original daily tweet volume. If *R*_*p*_(*τ*) = 1, the time series has strongly increasing or decreasing tendency with period of *τ*. If *R*_*p*_(*τ*) = −1, the time series shows ‘up and down’ or zigzag pattern with period of *τ*. If *R*_*p*_(1) ≈ 0.0, then we can consider *V*_*p*_(*t*) such that *V*_*p*_(*t*) = *V*_0_ + *E*_*t*_ where *V*_0_ is a constant and *E*_*t*_ is an error (or noise) term as described above. As shown in Fig 3, we observed positive autocorrelations *R*_*p*_(1) ≥ 0.2 for all of the cases. This means the daily tweet volume for parties have some ‘increasing’ or ‘decreasing’ patterns for some time intervals and cannot be described by a simple constant plus error model. However, *R*_*p*_(*τ* ≥ 2) ≈ 0 in some cases. In these cases the tendency do not last long. While *R*_*p*_(*τ* ≥ 2) ≥ 0.4 for M5S and AET in *Euro14* (Fig 3(A)), for M5S in *Italy13* (Fig 3(B)), and for DPS and ATAKA in *Bulgaria13* (Fig 3(C)). These cases show more persistent tendency.

### A model of fluctuations in tweet volume

The observed log-normal distributions of daily tweet volumes for parties suggest that its underlying dynamics can be described by a geometric Brownian motion (GBM) [38]. This means that the logarithm of the variable follows a Brownian motion with a drift, a situation that often describes the dynamics of company prices in stock markets [33].

To verify this assumption we need to check if the logarithmic ratio *r*_*p*_(*t*) = *log*(*V*_*p*_(*t* + 1)/*V*_*p*_(*t*)) follows a normal distribution and if the same ratio is independent of time [33, 39].

**Fig 4:**
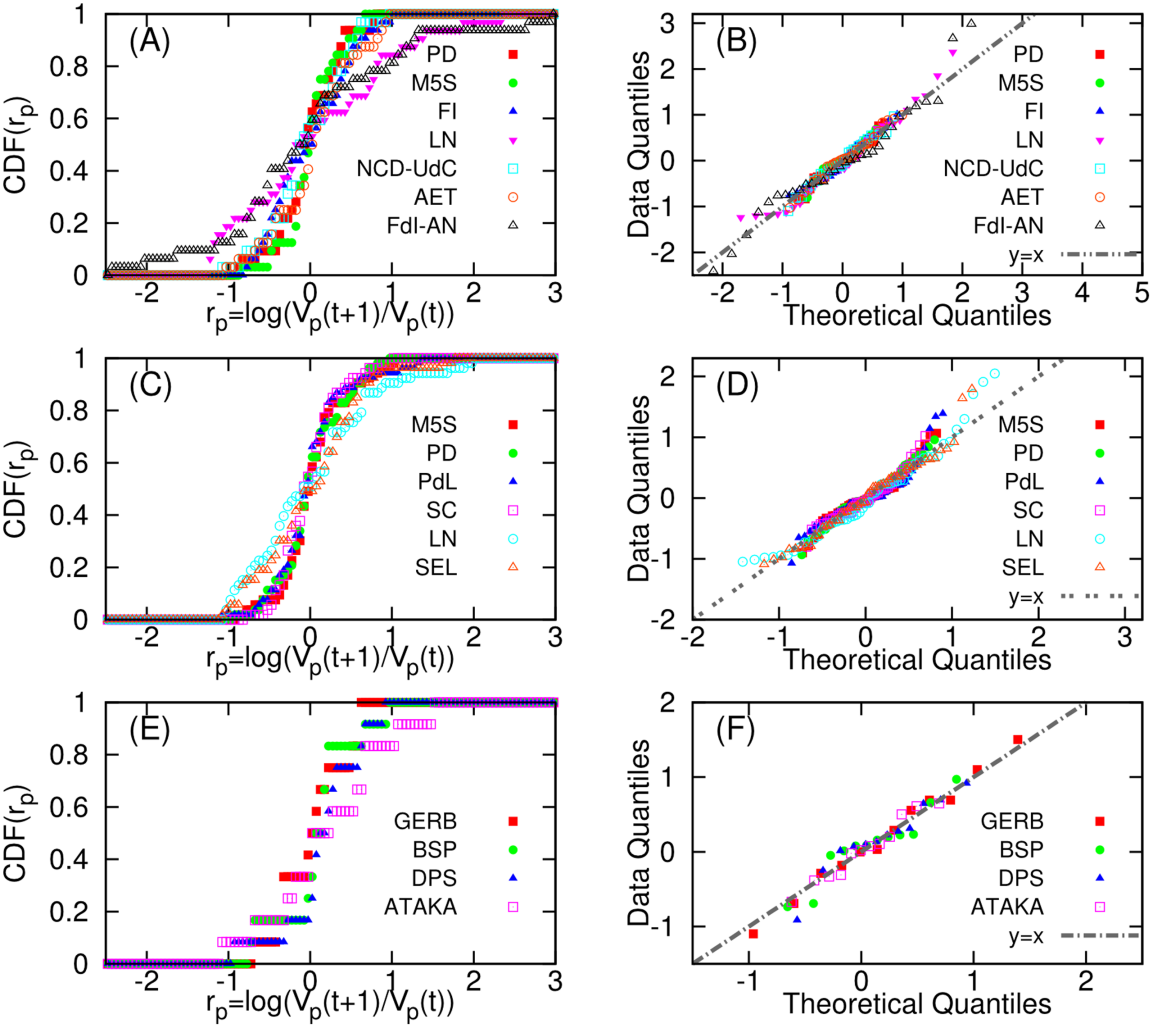
Normality of the logarithmic ratio *r*_*p*_(*t*) = *log*(*V*_*p*_(*t* + 1)/*V*_*p*_(*t*)) of two consecutive tweet volumes of party *p*. Cumulative distribution functions of the log ratio for each party are represented in (A) *Euro14*. (C)*Italy13*. (E) *Bulgaria13*. The Q-Q plots of the log ratio *r*(*t*) for each party are also represented in (B)*Euro14*. (D) *Italy13*. (F) *Bulgaria13*. The theoretical quantile is based on normal distribution. In the Q-Q plot, if the points lie on *y* = *x*, it means the log ratio follow a normal distribution.

**Fig 5:**
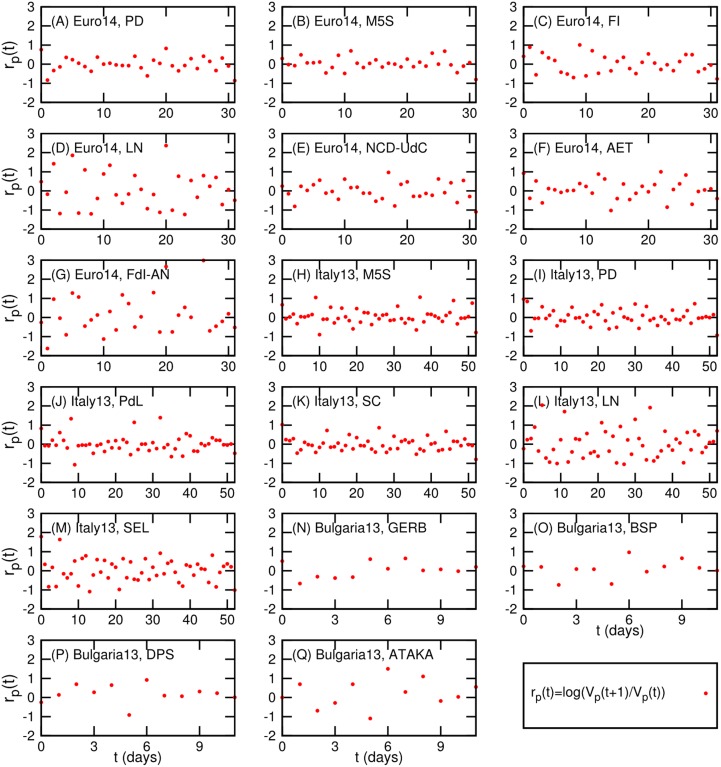
Scatter plot of time *t* and log ratio *r*_*p*_(*t*) = *log*(*V*_*p*_(*t* + 1)/*V*_*p*_(*t*)) for each party *p*. Here *V*_*p*_(*t*) is the tweet volume of the party *p* at time *t*.

Regarding the first point, we show in Fig 4(A), 4(C), and 4(E) the cumulative distribution functions of *r* for every party. To confirm that they are indeed normally distributed, we consider the Q-Q plots for each party as shown in Fig 4(B), 4(D), and 4(F) (as described in Fig 2). The Q-Q plots strongly support the normality of the logarithmic ratio *r*_*p*_(*t*) (the points approximately lie on *y* = *x* line). As for the second point we consider the scatter plots of the logarithmic ratio *r*_*p*_(*t*) = *log*(*V*_*p*_(*t* + 1)/*V*_*p*_(*t*)) as shown in Fig 5. From Fig 5 we can see that the ratio *r*_*p*_(*t*) for every party is independent of time *t*.

By fulfilling the above hypotheses, we can consider Eq 4 as a GBM model for dynamics of *V*_*p*_(*t*). By linear fitting of the data with Eq 4, we can determine the value of $\mu -\frac{\sigma^{2}}{2}$ and *log*(*V*_*p*_(0)). Then we get the value of *σ* from the fluctuations between the data and the GBM model. The obtained values of *μ*, *σ*, and *V*_0_ are represented in Table 3.

**Table 3:** Parameters to describe the dynamics of daily tweet volume of political parties as a geometric Brownian motion (GBM). The expectation value *V*_*p*_(*t*) of daily tweet volume of party *p* at time *t* given by a GBM is *V*_*p*_(*t*) = *V*_*p*_(0)*exp*((*μ*−*σ* ^2^/2)*t* + *σW*(*t*)) where *W*(*t*) is a Wiener process or a Brownian motion.

**Euro14: European Parliament election 2014, Italy**											
**Rank**	**Party**	*μ*−*σ* ^2^/2	*μ*	*σ*	*V*_*p*_(0)	**Rank**	**Party**	*μ*−*σ* ^2^/2	*μ*	*σ*	*V*_*p*_(0)
1	PD	0.0124	0.0627	0.3171	18299.2	5	NCD-UdC	0.0059	0.0893	0.4088	2578.5
2	M5S	0.0469	0.0925	0.3018	6143.3	6	AET	0.0581	0.1513	0.4316	520.0
3	FI	0.0053	0.0955	0.4247	9714.3	7	FdI-AN	0.0404	0.4013	0.8496	238.5
4	LN	0.0592	0.2995	0.6932	686.2						
**Italy13: Italian general election 2013**											
**Rank**	**Party**	*μ*−*σ* ^2^/2	*μ*	*σ*	*V*_*p*_(0)	**Rank**	**Party**	*μ*−*σ* ^2^/2	*μ*	*σ*	*V*_*p*_(0)
1	M5S	0.0328	0.0979	0.3608	3294.9	4	SC	-0.0048	0.0490	0.3278	22856.7
2	PD	0.0181	0.0815	0.3561	9121.2	5	LN	0.0104	0.2264	0.6573	576.0
3	PdL	0.0039	0.1164	0.4744	16763.5	6	SEL	0.0127	0.1406	0.5057	2458.3
**Bulgaria13: Bulgarian general election 2013**											
**Rank**	**Party**	*μ*−*σ* ^2^/2	*μ*	*σ*	*V*_*p*_(0)	**Rank**	**Party**	*μ*−*σ* ^2^/2	*μ*	*σ*	*V*_*p*_(0)
1	GERB	0.0435	0.1591	0.4808	194.8	3	DPS	0.2110	0.2496	0.2782	7.6
2	BSP	0.0892	0.1904	0.4498	55.8	4	ATAKA	0.2248	0.4020	0.5954	2.4

**Fig 6:**
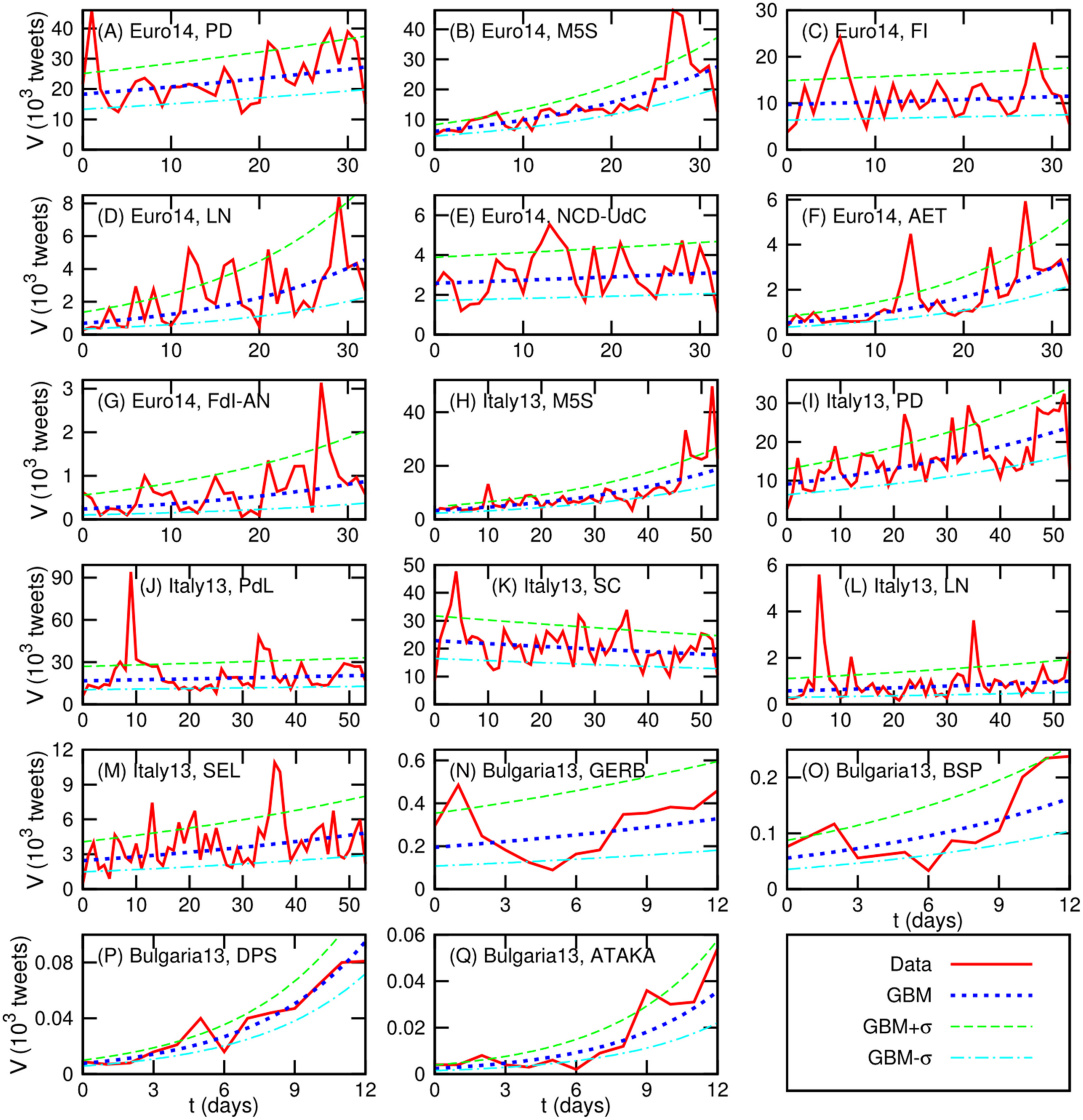
Dynamics of daily tweet volume for each party represented by data and by the GBM model. In the GBM model, the expected volume *V*(*t*) at time *t* is given by $V_{p}(t)=V_{p}(0)exp((\mu -\frac{\sigma^{2}}{2})t)$. In the GBM+*σ* model, $V_{p}(t)=V_{p}(0)exp((\mu -\frac{\sigma^{2}}{2})t+\sigma )$ while $V_{p}(t)=V_{p}(0)exp((\mu -\frac{\sigma^{2}}{2})t-\sigma )$ in the GBM−*σ* model. The values of parameters *μ*, *σ*, and *V*(0) are given in Table 3.

Fig 6 shows the dynamics of *V*_*p*_(*t*) for each party *p* (red lines) and the corresponding GBM model $V_{p}(0)exp((\mu -\frac{\sigma^{2}}{2})t)$ (blue dashed lines). As guidelines, GBM+*σ* model $V_{p}(0)exp((\mu -\frac{\sigma^{2}}{2})t+\sigma )$ (green dashed lines) and GBM−*σ* model $V_{p}(0)exp((\mu -\frac{\sigma^{2}}{2})t-\sigma )$ (cyan dashed lines) are also represented in Fig 6. Indeed, the GBM model describes well the dynamics of daily tweet volume in the data as shown in Fig 6 although there are some large spikes, which are beyond the GBM+*σ* model, in the dynamics. Also the obtained values of *μ* and *σ* explain the observed strong autocorrelations of daily tweet volumes. For example, M5S in *Euro14* and *Italy13* has relatively high *μ* but low *σ*, thus the dynamics of daily tweet volume of M5S in *Euro14* and *Italy13* has relatively strong drift with weak fluctuations. This leads the dynamics to high autocorrelations in longer term (i.e., a strong tendency with low volatility).

### Tweet volumes and election outcomes

Until now we mainly focused on the dynamical properties and the modelling of daily tweet volumes of political parties in order to describe the properties of data fluctuations. Anyhow, the simplest way of reducing fluctuations will be averaging out (or cumulating) the daily tweet volumes. However, positive autocorrelation and short-term memory of the volumes imply that if we consider too long time interval for averaging, we might lose short term increasing or decreasing tendency in the dynamics. In other words, if we consider too long period, the recent relevant signals from tweet volumes can be hidden by old tweet volumes. In addition, if we consider tweet volume in days much earlier than the election day, other types of ‘noise’ compromise the ‘signal’. Twitter users typically do not pay much attention to elections before the campaign actually starts, even though they may mention “politics” in their tweets. Thus it is necessary to find out how long time interval has to be considered to get optimal results in practical sense.

To identify the optimal time interval of averaging daily tweet volume of a given political party, we consider the tweet volume ${\bar{V}}_{p}(\lambda )$ of a party *p* averaged from the day before the election to the |*λ*| days before as follows:

$${\bar{V}}_{p}\left(\lambda \right)=\frac{1}{\left|\lambda \right|}\sum_{t=t_{e}-\left|\lambda \right|}^{t_{e}-1}V_{p}\left(t\right).$$ (6)

Here *t*_*e*_ is the election day, *λ* is a negative integer, and |*λ*| is the absolute value of *λ* that represents the number of days to wait for the election day (i.e., *λ* = −2 means two days before the election day).

**Fig 7:**
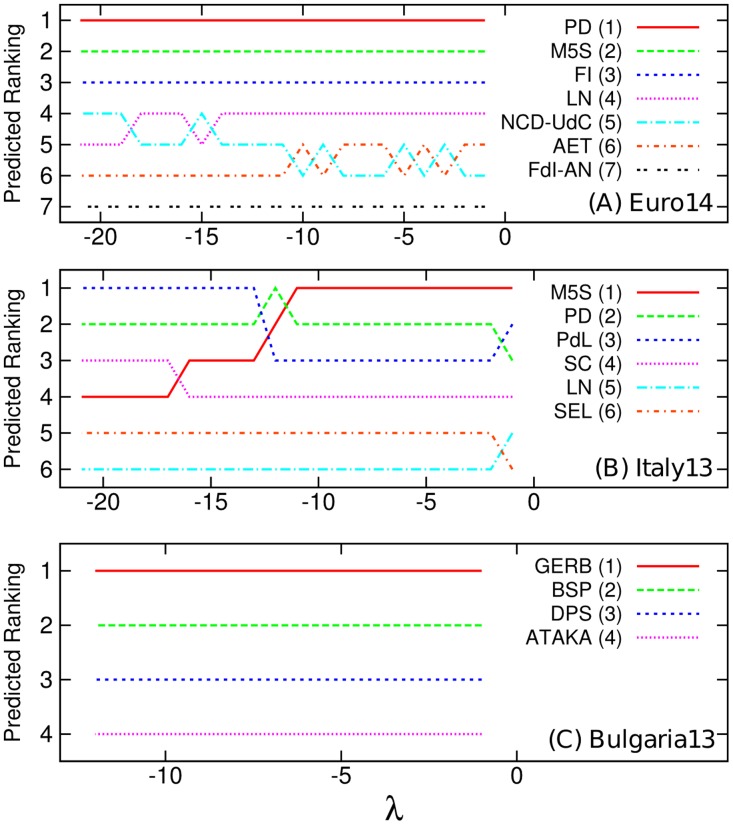
Predicted ranking determined by tweet volume ${\bar{V}}_{p}(\lambda )$ averaged from the day before the election to the *τ* days before the election. ${\bar{V}}_{p}(\lambda )$ is given by Eq 6. The numbers in parentheses represent actual rankings of the parties in the election. (A)*Euro14*. (B) *Italy13*. (C) *Bulgaria13*.

Fig 7 shows the rankings of parties ordered by ${\bar{V}}_{p}(\lambda )$ for each time interval from the day before the election day to the |*λ*| days before the election. For the case of *Euro14* (Fig 7(A)), until *λ* = −14, we can get the accurate prediction. For the case of *Italy13*, the optimal length of time interval for accurate prediction will be from *λ* = −2 to *λ* = −11. Indeed, M5S performed much better than the expectation before the election and the support for M5S was rapidly growing during the campaign. This pattern is vividly reflected in Fig 7(B). If we consider *λ* = −14, then the prediction based tweet volume M5S anticipated M5S will be the third thanks to the low supports for M5S in earlier period of the campaign. On the other hand, all considered *λ* show accurate and consistent prediction in the case of *Bulgaria13* (Fig 7(C)), as expected from Fig 1.

## Discussion

Social media permeate all levels of society rapidly and widely. A huge amount of data on collective behaviors are being generated from these social media. This phenomenon promotes quantitative analysis of these data, with the goal to understand collective behaviors and predict them in effective and efficient ways. In this paper, we analyzed dynamics of daily tweet volumes of political parties on Twitter, when approaching elections, identified statistical patterns of the daily tweet volumes of parties, and described the dynamics of volume with geometric Brownian motion (GBM). We found that the daily tweet volume of a given political party follows a broad distribution like log-normal, and has positive autocorrelation over a short time period. Finally, we identified there is an optimal period of averaging tweet volumes which not only reduce the fluctuation but also keep the short term tendency of tweet volumes. Our analysis shows that daily tweet volumes could have a limited prediction ability of election outcomes and that this limitation is caused by their strong fluctuations.

In order to overcome the limited prediction power of the daily tweet volume, one needs to understand what causes statistical fluctuations of Twitter activity and to separate the signal from the noise in tweet volumes. Universal features of fluctuations with the form of log-normal distributions imply that there might be a single underlying mechanism for the fluctuations, such as multiplicative processes [32]. In particular, the driving mechanisms of peaked activities, which cause large fluctuations, should be understood. For instance, Silvio Berlusconi is a popular figure in Italian politics and society. He therefore receives a large number of Twitter mentions not only by his supporters but also by his opponents; often these mentions are not just about politics but also about his private life. For example, on 9 Jan. 2013, a sharp peak of FI (i.e., mentioning Berlusconi) in Fig 1 was observed. From the news on this day we concluded that an Italian court fixed the financial consequences of his divorce and that he was charged with the accusation of prostitution with a minor (at the time of publication of this article the trial ended and he was sentenced not guilty). This example clearly illustrates that the peaks could stem not only from election issues but also from private issues of the politicians. This also means that one needs to consider the roles of mass media for daily tweet volumes of political parties. All these factors can have significant influence on tweet volumes of political parties or politicians. Systemic consideration of these factors can give us some hints about the amount of the fluctuations originating from the endogenous or exogenous mechanisms.

Expanding the point of view, it would be interesting to identify whether the dynamics after the election also can be described as a GBM or not. If possible, the GBM model for the dynamics after the election might have different drift (*μ*) and volatility (*σ*) terms in Eq 4 from the ones in the current GBM model for the dynamics before the election. Because, as shown in Fig 1, the dynamics of tweet volume typically shows a peak on the election day or the day after election and show decreasing patterns hereafter. This implies the drift (i.e., tendency) term of the GBM might be changed after the election since the collective attention was moved to other issues. In order to describe the dynamics after election as a GBM, it is necessary to test the normality of logarithmic ratio of consecutive tweet volume and time-independence of the ratio as done in Figs 4 and 5. For these tests, we need to consider tweets data-set collected after the elections.

Not only single social media but also multiple social media can be considered to predict the election outcome. For instance, Wikipedia and search engine data have been used to forecast elections outcomes [31], and sentiment analysis was suggested for reinforcing the forecasting performance. Checking the validity of combined social media data will be one of our future research directions.

Another interesting problem worth to be considered is to determine if the patterns of daily tweet volumes of political parties (for example, log-normal distribution) have universal features. If this is the case, it would be important to determine if we observe similar patterns for other events. Indeed, broad distributions of tweet volume for brand names [42] and attentions to online items [43] have already been reported. Hence, investigation of dynamics of tweet volumes of various objects can lead us to check universal features of the dynamics. Further research will be necessary to determine this point.

Influence of social media on political and social issues is getting greater and greater. Understanding mathematical nature of dynamics of collective attention to elections in social media can enhance our ability to anticipate dynamics of collective attention to other political or social issues.
